# Ultra-processed food consumption by children from a Pelotas Birth Cohort

**DOI:** 10.11606/s1518-8787.2022056003822

**Published:** 2022-08-22

**Authors:** Anna Müller Pereira, Romina Buffarini, Marlos Rodrigues Domingues, Fernando Celso Lopes Fernandes Barros, Mariângela Freitas da Silveira

**Affiliations:** I Universidade Federal de Pelotas Faculdade de Medicina Programa de Pós-Graduação em Epidemiologia Pelotas RS Brasil Universidade Federal de Pelotas. Faculdade de Medicina. Programa de Pós-Graduação em Epidemiologia. Pelotas, RS, Brasil; II Universidade Federal de Pelotas Escola Superior de Educação Física Programa de Pós-Graduação em Educação Física Pelotas RS Brasil Universidade Federal de Pelotas. Escola Superior de Educação Física. Programa de Pós-Graduação em Educação Física. Pelotas, RS, Brasil

**Keywords:** Infant Nutrition, Infant Food, Industrialized Foods, Child Development, Cohort Studies

## Abstract

**OBJECTIVE:**

Assessing the regular consumption of ultra-processed foods by children at 24 months of age from the 2015 Pelotas Birth Cohort and the main demographic, socioeconomic, and behavioral factors related to the consumption of these products.

**METHODS:**

Population-based cohort in the city of Pelotas, RS, where 4,275 children were assessed at birth and 95.4% of them were followed up until 24 months of age. Food consumption was assessed by a questionnaire on regular consumption of ultra-processed foods, which collected information regarding sex, household income, maternal skin color, schooling level, and age, the child attending day care and having siblings, breastfeeding status, and obesity. The outcome was the sum of ultra-processed foods regularly consumed by a child. A multivariate Poisson regression analysis was used to calculate the association between the regular consumption of ultra-processed foods and exposure variables.

**RESULTS:**

The mean number of ultra-processed foods consumed was 4.8 (SD = 2.3). The regular consumption of ultra-processed foods was positively associated with black skin color and having siblings, and negatively associated with household income and maternal schooling level and age.

**CONCLUSION:**

The mean regular consumption of ultra-processed foods by children from the 2015 Pelotas Birth Cohort is high, which can negatively affect the children’s diet. The risk of consuming this kind of food was higher among children from families of lower socioeconomic status, whose mothers present lower education level, black skin color, and younger age.

## INTRODUCTION

In the last few years, the lifestyle of Brazilian population and its eating behavior underwent transformations. Natural or minimally processed foods have been replaced by processed and ultra-processed foods (UPFs), which are being introduced earlier and earlier in infant feeding^1 , 2^. This scenario contributes to the imbalance in the supply of nutrients and the high consumption of high-calorie foods, favoring the development of obesity, which, in the child population, is related to the early weaning and the introduction of unhealthy foods in complementary feeding^3^.

According to the NOVA classification, by Monteiro et al.^4^, ultra-processed foods are products that undergo various stages and processing techniques and receive additives, such as sweeteners, food coloring, flavorings, and flavor enhancers, in order to make the products more attractive and extend their shelf life. These products are nutritionally unbalanced, as they present high energy density, high fat density, high sugar and sodium content, and little fiber and protein content^4^, and must be avoided before the first two years of a child’s life, since the excessive consumption of these foods is associated with anemia, overweight, and food allergies^5^, besides presenting long-term effects on children’s health, making them prone to develop chronic diseases in adulthood^3^.

The evolution of household food availability in Brazil, calculated according to the *Pesquisas de Orçamentos Familiares* (POF – Consumer Expenditure Surveys), carried out in the intervals 2002–2003, 2008–2009, and 2017–2018, shows that natural or minimally processed foods have lost ground to processed foods and, especially, ultra-processed foods^6^. In Pelotas, dietary data from participants of the 1982, 1993, and 2004 birth cohorts showed that the daily energy contribution of ultra-processed foods was higher within the younger cohort (25.1%, 29.8%, and 33.7% at ages 30, 22, and 11, respectively)^7^.

A study carried out with children aged six to 12 months in a city in the metropolitan region of São Paulo, which assessed the ultra-processed food consumption in the last 24 hours before the interview, showed that the prevalence of consumption of these foods was 43% and children who were not being breastfed, as well as children born to mothers with lower schooling level, present higher prevalence of consumption of these foods^8^. Another study, carried out with children up to 72 months of age from the city of Pelotas, RS, showed that the mean daily energy consumption was 1,725 kcal/day and the calorie intake of ultra-processed foods was 19.7% for children under 24 months of age and 36.1% for those aged 24 months or older^9^. However, data on the ultra-processed food consumption by preschool children are still emerging.

The first two years of a child’s life are fundamental for the encouragement and adoption of healthy eating habits, as well as for the prevention of chronic diseases in later stages of life, since the eating habits established at this stage of life tend to remain and consolidated in adulthood^10^. Therefore, assessing the ultra-processed food consumption by children who are in the introduction phase of complementary feeding is important, using a classification that not only considers the nutrients in a food, but also its degree of processing. It contributes to a better understanding of the factors associated with the ultra-processed food consumption, as well as with data that can help in the development of materials with guidance on eating practices, such as the new *Guia Alimentar para Crianças Brasileiras Menores de 2 Anos* (Dietary Guidelines for Brazilian Children Under 2 Years of Age) (2019)^5^, which considers food processing when recommending food for this age group.

This study aimed to assess the regular consumption of ultra-processed foods by children at 24 months of age from the 2015 Birth Cohort in the city of Pelotas, RS, and the demographic, socioeconomic, and behavioral factors related to this consumption.

## METHODS

### Type of Study and Sample

This is a cross-sectional study using data of the 2015 Birth Cohort carried out in Pelotas, a mid-sized city in Southern Brazil. It is a longitudinal study of all live births. All 4,333 children born to mothers living in the urban area of the city were eligible to participate in the study although, considering a loss and refusal rate of 1.3% and 54 stillbirths, the final sample of the 2015 Birth Cohort was 4,275 children. For the perinatal follow-up visit, interviews were performed from 24 to 48 hours after childbirth. Later, mothers and children were interviewed on several occasions (at three, 12, 24, and 48 months of age of children). The follow-up rate of the 2015 Birth Cohort of children aged 24 months was 95.4% (n = 4,014). More information is available in the 2015 Cohort profile^11^.

### Variables

The outcomes of this study were evaluated at 24 months of age of children and data was collected by a questionnaire with 20 questions related to the child’s diet, based on the following question: “ *Now, I am going to ask you a few questions about the <CHILD’S> diet. Please, answer based on the foods that are regularly consumed, it is, every or almost every day. Thinking about the <CHILD’S> usual consumption, he/she eats/drinks”* . Mothers should answer yes or no to this question.

The food consumption questionnaire used in the 24-month follow-up of the 2015 Birth Cohort was based on the food consumption markers form of the *Sistema de Vigilância Alimentar e Nutricional* (SISVAN – Food and Nutrition Surveillance System)^12^. Some adjustments were made, based on the empirical knowledge about birth cohorts and consumption patterns of the inhabitants of Pelotas, especially in relation to the examples of the items.

The food items were classified according to the NOVA classification^4^ and ultra-processed foods were separated into nine groups: instant noodles, soft drinks, boxed or bottled juice, powdered juice or boxed coconut water, nuggets, hamburger, or processed meats (such as ham, mortadella, salami, sausage and hot dogs), package snacks (like chips), yogurt, sweet biscuits or stuffed cookies, candies, lollipops, chewing gums, chocolate, or jelly, and chocolate milk. The outcome was the sum of ultra-processed foods regularly consumed by a child, ranging from zero to nine.

The independent variables collected during the perinatal follow-up were sex of the child, maternal skin color (white, black, or brown), household income in Brazilian currency (the sum of the individual income of all residents of the house categorized into quintiles), maternal age in complete years of age (< 20, 20–34, and ≥ 35), maternal schooling level in complete years of formal education (0–4, 5–8, 9–11, and ≥ 12), and “having siblings” (no/yes). The variable breastfeeding status (weaned or partially weaned) was collected at 12 months of age of the child and the variable “attending nursery” (no/yes) was collected at his or her 24 months of age^a^.

### Data Analysis

To characterize the study sample, a descriptive analysis was performed with absolute (n) and relative (%) frequency of the ultra-processed food consumption and the independent variables. The sum of the regular consumption of ultra-processed foods by children at 24 months of age is also presented. The association between the number of ultra-processed foods regularly consumed and the independent variables was performed using crude and adjusted Poisson regression analyses. Relative risks and their respective 95% confidence intervals (95%CI) were calculated for each predictor presented. Analyses were performed following a three-level hierarchical model, the first level composed of the variables: sex, maternal skin color, age, and schooling level, and household income; the second level composed of the variables: child attending nursery and having siblings; and the third level composed of the variable “breastfeeding status”. Independent variables from the same level were inserted in the regression at the same time. The variables that presented p < 0.20 remained in the adjusted model and, thus, other variables were added for each level. Statistical significance was p < 0.05. Analyses were performed in Stata 15.1 software.

### Ethical Aspects

The study protocol was approved by the Research Ethics Committee of the *Escola Superior de Educação Física* of *Universidade Federal de Pelotas* (CAAE registration number: 26746414.5.0000.5313) and all participants signed an informed consent form before each interview.

## RESULTS

Table 1 presents the demographic, socioeconomic, and behavioral characteristics of the sample. Slightly more than half of the sample were boys (50.6%). In about two-thirds of the sample, mothers were white (70.9%) and aged from 20 to 34 years (70.6%). One-third of mothers presented from nine to 11 years of schooling (34.1%) and about half of the children (50.9%) had no siblings. At 12 months of age, 58.8% of the children were already weaned and more than two-thirds did not attend nursery at 24 months of age.

**Table 1:** Description of the sample. Pelotas Birth Cohort-RS, 2015.

Variable	n (%)
Sex	
Women	2,111 (49.4)
Men	2,164 (50.6)
Maternal skin color	
White	3,024 (70.9)
Black	667 (15.6)
Brown	551 (12.9)
Household income^a^	
Q1 (poorer)	846 (19.8)
Q2	859 (20.1)
Q3	853 (19.9)
Q4	856 (20.0)
Q5 (richer)	859 (20.1)
Maternal schooling level	
0–4 years of schooling	391 (9.2)
5–8 years of schooling	1,095 (25.6)
9–11 years of schooling	1,458 (34.1)
≥ 12 years of schooling	1,330 (31.1)
Maternal age	
< 20 years old	623 (14.6)
20–34 years old	3,018 (70.6)
≥ 35 years old	633 (14.8)
The child attends nursery at 24 months of age	
No	2,940 (73.3)
Yes	1,071 (26.7)
The child has siblings	
No	2,174 (50.9)
Yes	2,100 (49.1)
Breastfeeding status until the child is 12 months of age	
Partial breastfeeding	1,653 (41.2)
Weaned	2,359 (58.8)

Q: quintile; m: mean; DP: standard deviation.

^a^ Household income: Q1 (m = 725.17; SD = 290.42); Q2 (m = 1,369.10; SD = 178.70); Q3 (m = 2,038.80; SD = 211.64); Q4 (m = 3,011.86; SD = 379.93); Q5 (m = 8,133.88; SD = 7,702.16).

Figure shows the sum of ultra-processed foods consumed by children at 24 months of age and Table 2 presents the prevalence of regular consumption of each ultra-processed food group. A total of 4.6% of the children regularly consumed foods from the nine groups. The mean regular consumption of UPFs by children at 24 months of age was 4.8 foods, and the median was five foods. (SD = 2.3; P25: 3; P75: 7). The prevalence of regular consumption in each ultra-processed food category ranged from 29.6% to 88.3%. The least consumed food was instant noodles, followed by soft drinks, and the most consumed ultra-processed food was yogurt.

**Figure:**
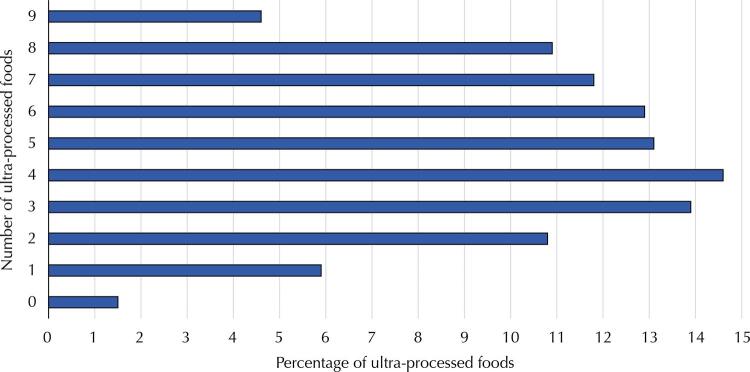
Frequency (%) of regular consumption of ultra-processed foods by children at 24 months of age. Pelotas Birth Cohort-RS, 2015.

**Table 2:** Prevalence of regular consumption of ultra-processed foods by children at 24 months of age. Pelotas Birth Cohort-RS, 2015.

Ultra-processed foods	n (%)
Boxed or bottled juice, powdered juice, or boxed coconut water	2,641 (65.8)
Soft drinks	1,498 (37.4)
Instant noodles	1,188 (29.6)
Nuggets, hamburger, or processed meats	1,728 (43.1)
Packaged snacks (like chips)	1,844 (46.0)
Yogurt	3,542 (88.3)
Sweet biscuits or stuffed cookies	2,587 (64.5)
Candies, lollipops, chewing gums, chocolate, or jelly	2,582 (64.4)
Chocolate milk	1,724 (43.0)

Table 3 shows the association between the regular consumption of ultra-processed foods and possible risk factors for children at 24 months of age. The variables household income, maternal skin color, schooling level, and age, “attending nursery”, and “having siblings” were associated in the crude analysis and remained so in the adjusted analysis, except for the variable “attending nursery”, which lost its association after adjustment for potential confounders ( Table 3 ).

**Table 3:** Regular consumption of ultra-processed food by children at 24 months of age and associated factors. Pelotas Birth Cohort-RS, 2015.

	Crude			Adjusted^a^		
	
	RR	p	95%CI	RR	p	95%CI
Sex		0.686			0.919	
Men	0.99		0.97–1.03	1.00		0.97–1.03
Women	ref			ref		
Maternal skin color		< 0.001			< 0.001	
White	ref			ref		
Black	1.26		1.21–1.30	1.12		1.08–1.16
Brown	1.21		1.16–1.26	1.07		1.03–1.12
Household income		< 0.001			< 0.001	
Q1 (poorer)	1.67		1.59–1.75	1.19		1.12–1.26
Q2	1.54		1.47–1.62	1.17		1.11–1.23
Q3	1.44		1.37–1.51	1.16		1.10–1.22
Q4	1.34		1.27–1.40	1.15		1.09–1.21
Q5 (richer)	ref			ref		
Maternal schooling level (years of schooling)		< 0.001			< 0.001	
0–4	1.83		1.74–1.92	1.59		1.50–1.69
5–8	1.69		1.62–1.75	1.47		1.40–1.54
9–11	1.44		1.39–1.50	1.31		1.26–1.37
≥ 12	ref			ref		
Maternal age (years old)		< 0.001			< 0.001	
< 20	1.54		1.46–1.62	1.23		1.17–1.30
20–34	1.22		1.16–1.27	1.14		1.09–1.19
≥ 35	ref			ref		
The child attends nursery		< 0.001			0.429	
No	1.16		1.12–1.20	0.99		0.95–1.02
Yes	ref			ref		
The child has siblings		< 0.001			< 0.001	
No	ref			ref		
Yes	1.11		1.08–1.14	1.10		1.06–1.13
Breastfeeding status until the child is 12 months of age		0.242			0.104	
Partial breastfeeding	ref			ref		
Weaned	1.02		0.99–1.05	1.02		1.00–1.06

RR: relative risk; 95%CI: 95% confidence interval.

^a^ Adjustment by hierarchical level: level 1: sex, maternal skin color, household income, maternal schooling level, and maternal age; level 2: the child attends nursery and the child has siblings; level 3: breastfeeding status until the child is 12 months of age.

The mean number of ultra-processed foods consumed by children whose mothers had black and brown colour, was 12% and 7% higher when compared with the mean number of ultra-processed foods consumed by children with white mothers, respectively. Household income was associated with regular consumption of ultra-processed foods, showing that the risk of consuming these foods is higher in children from families in the poorest income quintile (RR = 1.19; 95%CI: 1.12–1.26). Maternal schooling level showed a negative tendency towards an association with the regular consumption of ultra-processed foods, showing that children born to less educated mothers present higher risks of regularly consuming ultra-processed foods (RR = 1.59; 95%CI: 1.50–1.69). Similarly, the younger the mother, the higher the mean number of ultra-processed foods consumed. Children with siblings presented a mean number of ultra-processed foods consumed 10% higher than children without siblings.

## DISCUSSION

This study assessed the regular consumption of ultra-processed foods by children at age of 24 months from the 2015 Birth Cohort in the city of Pelotas, RS; and demographic, socioeconomic, and behavioral factors associated with this consumption. The high mean number of ultra-processed foods consumed and the early introduction of UPFs in infant feeding found in our study is in accordance with the literature, although the assessment methods vary among studies^3 , 8^. In Giesta et al.^3^(2017), only 21% of a sample of children aged four to 24 months from the city of Porto Alegre, RS, had not yet consumed any type of UPF, and 56% consumed some of these foods before six months of age. In Relvas et al.^8^ (2019), 43% of a sample of children aged six to 12 months from a city in the metropolitan region of São Paulo, SP, had consumed at least one type of ultra-processed food in the previous 24 hours of interview. Besides the practicality, convenience, and high palatability, the exposure of children to advertisements that highlight high-calorie foods, such as ultra-processed foods, has been contributing to develop the recollection and familiarity with foods and brands (by labels and packages), which can encourage and influence their purchase and consumption^13^.

Th excessive consumption of ultra-processed foods is consistently associated with a general deterioration of the nutritional quality of diets, besides favoring the excessive calorie consumption^14^. Moreover, the early exposure to sweeteners (both caloric and non-caloric), used in the preparation of ultra-processed foods, can cause adverse effects on the body composition, cardiometabolic health, and gut microbiota of children, besides creating preference for sweetness, which influences eating habits from childhood to adulthood^15^. The data found in our study are alarming, considering the strong evidence that ultra-processed foods must bet avoided in the first years of a child’s life and consumed with restrictions in all other stages, favoring the development of healthy eating habits and, consequently, the prevention of chronic non-communicable diseases^10 , 14^.

In recent decades, the ultra-processed food consumption increased within Brazilian families. Data from the last three editions of the *Pesquisa de Orçamentos Familiares* ^6^show that UPFs represented 12.6% of daily calories from 2002 to 2003, 16% from 2008 to 2009, and 18.4% of total calories in the last edition (from 2017 to 2018). Eating habits are influenced by biological, nutritional, cultural, and socioeconomic factors and, for children, besides these factors, diet is also influenced by the habits of their parents and family^16^. Thus, due to the increasing presence of ultra-processed foods in the Brazilian diet, the early introduction of this type of food in infant feeding becomes usual, affecting eating practices, health status and nutrition throughout life.

Among children from the 2015 Birth Cohort, those from the poorest and least educated families presented higher risks of regularly consuming ultra-processed foods. National data show that the consumption of these foods increased in all social strata, however, this increase was higher in classes with lower income^6^. In developing countries, such as Brazil, the nutrition transition usually occurs first among individuals with higher socioeconomic status. However, the advance of development and progression of the nutrition transition allow greater access to unhealthy foods for groups with lower socioeconomic status^17^. In our study, Black and mixed-race children presented higher mean number of ultra-processed foods consumed when compared to white children. Considering that skin color is a marker of economic level, it may be related to the increased access of the population with lower socioeconomic status to UPFs, since, besides presenting longer shelf life, these foods generally cost less than fresh foods, such as meats, fruits, and vegetables^18^. UPFs are more practical foods, which require no or almost no preparation and can be consumed at any time, anywhere, and it contributes to the preference for their purchase^19^. In this sense, public policies such as the *Programa Nacional de Alimentação Escolar* (PNAE – National School Feeding Program)^20^become important, aiming to offer food, promote food and nutrition education actions to all students from public basic education schools, and address nutritional and dietary shortcomings of children who lack access to quality food.

The literature also shows that, besides the low income and maternal schooling level, the presence of siblings in the family composition is also a socioeconomic predictor of a diet rich in ultra-processed foods^21 , 22^. Among children from the 2015 Birth Cohort, those with siblings present a higher mean number of UPFs consumed when compared to only children.

The regular consumption of ultra-processed foods was associated with low maternal schooling level, as presented by previous studies^8 , 23^. A national survey presented an association between the high frequency of consumption of unhealthy foods by children under one year of age and the low maternal schooling level^23^. Similarly, Relvas et al.^8^ (2019) showed that the highest consumption of ultra-processed foods by children aged six to 12 months was among those born to less educated mothers. Maternal age was negatively associated with the regular consumption of ultra-processed foods, since children born to younger mothers presented higher risks of regularly consuming ultra-processed foods, in accordance with the literature^24 , 25^, which shows that younger mothers are prone to early introduce this type of food in infant feeding.

Less access to information on healthy eating practices—due to either lack of knowledge or lack of professional guidance in the first year of the child’s life—possibly explains why lower maternal schooling level and younger maternal age are associated with higher risks of consuming UPFs. Studies^3 , 26^ show that nutritional interventions are effective in reducing the UPF consumption, regardless of maternal age and schooling level, when performed with children in their first year of life, by nutritional guidelines based on the ten steps to healthy eating for Brazilian children under two years of age^5^. The *Estratégia Amamenta e Alimenta Brasil* (EAAB – Brazilian Breastfeeding and Feeding Strategy)^27^, which promotes and encourages breastfeeding and healthy complementary feeding in the Brazilian Unified Health System, is an important contribution to disseminate information on the importance of healthy eating in this stage of life. The new *Guia Alimentar para Crianças Brasileiras Menores de 2 Anos* ^5^, which provides information on the degree of food processing and guidelines to promote healthy eating in the first two years of a child’s life, is also a guiding tool for policies, programs, and actions aiming to support, protect, and promote the health and food and nutrition security of Brazilian children, helping to reduce the ultra-processed food consumption by children aged six to 24 months.

Our study presented limitations. The tool used for data collection did not allow us to estimate the energy consumption, since we did not know the amount of ultra-processed foods consumed, as the food questionnaire used in the 2015 Birth Cohort was qualitative and not quantitative. Thus, from the tool used, we could not adjust the UPF consumption by energy. However, some national health surveys, such as the *Pesquisa Nacional de Saúde* (PNS – National Survey of Health), *Pesquisa Nacional de Demografia e Saúde* (PNDS – National Demographic and Health Survey), and *Vigilância de Fatores de Risco e Proteção para Doenças Crônicas por Inquérito Telefônico* (VIGITEL – Surveillance System for Risk and Protective Factors for Chronic Diseases by Telephone Survey), use similar approaches to the one we used in this study, which questionnaires assess only the frequency of food consumption and not its quantity. The food questionnaire used in the 2015 Birth Cohort, although developed based on known tools, such as the one of the *Sistema de Vigilância Alimentar e Nutricional* ^12^, was not validated for children aged 24 months in Pelotas, RS. On the other hand, one of the strengths of our study was that data were collected from a population-based cohort study with a large sample size and a high follow-up rate of children up to 24 months of age, which allows the generalization of our results to mid-sized cities. Moreover, our study contributes to a better understanding of the factors associated with the ultra-processed food consumption, contributing to public policies focused on food and nutrition, such as the *Programa Nacional de Alimentação Escolar* (National School Feeding Program) and food guides.

This study showed that the mean regular consumption of ultra-processed foods by children from the 2015 Birth Cohort in the city of Pelotas, RS, is high and the risk of consumption of this kind of foods is higher among children born to mothers with lower schooling level, lower income, and younger age. According to the recommendations of the *Guia Alimentar para Crianças Brasileiras Menores de 2 Anos* ^5^, the UPF consumption must be avoided in this age group, since these foods are nutritionally unbalanced and have high caloric content, large amounts of saturated fat and sodium, and low fiber and protein content. These foods also contain many additives, which can cause adverse effects on children’s health.

Our results reinforce the need for preventive measures and food and nutrition education actions aimed at mothers with lower schooling level, lower income, and younger age and health professionals responsible for nutritional guidance, since childhood is an important period for encouraging and developing healthy eating practices. More studies regarding the ultra-processed food consumption by preschool children are still necessary. We suggest more research on the determinants of this consumption and interventions to change this scenario.

## Footnotes

a The questionnaires used in each follow-up of the 2015 Pelotas Birth Cohort are available at: http://epidemio-ufpel.org.br/site/content/coorte_2015/questionarios.php .
