# Supplementary material for: Ultra-processed food consumption by children from a Pelotas Birth Cohort
Source: Rev Saude Publica. 2022 Aug 22;56:79. doi: 10.11606/s1518-8787.2022056003822 (PMC9423094; doi:10.11606/s1518-8787.2022056003822)
Supplement: Translation

# Translation: Consumo de alimentos ultraprocessados por crianças de uma Coorte de Nascimentos de Pelotas

**Authors:** Anna Müller Pereira, Romina Buffarini, Marlos Rodrigues Domingues, Fernando Celso Lopes Fernandes Barros, Mariângela Freitas da Silveira

**Affiliations:** I Brasil Universidade Federal de Pelotas. Faculdade de Medicina. Programa de Pós-Graduação em Epidemiologia. Pelotas, RS, Brasil; II Brasil Universidade Federal de Pelotas. Escola Superior de Educação Física. Programa de Pós-Graduação em Educação Física. Pelotas, RS, Brasil

**Keywords:** Nutrição do Lactente, Alimentos Infantis, Alimentos Industrializados, Desenvolvimento Infantil, Estudos de Coortes

## Abstract

**OBJETIVO:**

Avaliar o consumo habitual de alimentos ultraprocessados aos 24 meses de idade por crianças pertencentes à Coorte de Nascimentos de Pelotas de 2015 e os principais fatores demográficos, socioeconômicos e comportamentais relacionados ao consumo desses produtos.

**MÉTODOS:**

Coorte de base populacional na cidade de Pelotas-RS, onde foram avaliadas 4.275 crianças ao nascimento, das quais 95,4% foram acompanhadas até os 24 meses. O consumo alimentar foi avaliado por meio de um questionário de consumo habitual de alimentos ultraprocessados, coletando informações sobre sexo, renda familiar, cor da pele, escolaridade e idade da mãe, frequentar creche, ter irmãos, status de amamentação e obesidade. O desfecho foi a somatória de alimentos ultraprocessados consumidos habitualmente pela criança. Análise multivariada por regressão de Poisson foi utilizada para estimar a associação entre consumo habitual de alimentos ultraprocessados e as variáveis de exposição.

**RESULTADOS:**

O número médio de alimentos ultraprocessados consumidos habitualmente foi de 4,8 (DP = 2,3). O consumo habitual de alimentos ultraprocessados foi associado positivamente à cor da pele preta e ter irmãos e negativamente associado com renda familiar, escolaridade e idade materna.

**CONCLUSÕES:**

A média de consumo habitual de alimentos ultraprocessados por crianças pertencentes à Coorte de Nascimentos de 2015 da cidade de Pelotas é elevada, o que pode causar um efeito negativo na dieta das crianças. O risco de consumo desses alimentos foi maior entre crianças de famílias de menor posição socioeconômica, filhas de mães de baixa escolaridade, de cor da pele preta, mais jovens e de baixa renda.

## INTRODUÇÃO

Nos últimos anos, o estilo de vida da população e seu comportamento alimentar sofreram transformações, alimentos *in natura* ou minimamente processados foram substituídos por alimentos processados e ultraprocessados (AUP), introduzidos cada vez mais precocemente na alimentação infantil^1 , 2^. Tal cenário contribui para o desequilíbrio na oferta de nutrientes e para o alto consumo de alimentos calóricos, favorecendo o desenvolvimento da obesidade que, na população infantil, está relacionada com o desmame precoce e com a introdução de alimentos não saudáveis na alimentação complementar^3^.

De acordo com a classificação NOVA, proposta por Monteiro et al.^4^, os alimentos ultraprocessados são produtos que sofrem diversas etapas e técnicas de processamento e recebem aditivos como edulcorantes, corantes, aromatizantes e realçadores de sabor, utilizados para deixar os produtos mais atraentes e com maior durabilidade nas prateleiras. Esses produtos são nutricionalmente desequilibrados, pois possuem alta densidade energética, alta densidade de gordura, alto teor de açúcar e sódio e pouco teor de fibras e proteínas^4^, e devem ser evitados antes dos dois anos de vida, uma vez que o consumo excessivo desses alimentos está associado à anemia, ao excesso de peso e a alergias alimentares^5^, além de ter efeitos a longo prazo na saúde das crianças, predispondo-os ao desenvolvimento de doenças crônicas na vida adulta^3^.

A evolução da disponibilidade domiciliar de alimentos no Brasil, estimada com base nas Pesquisas de Orçamentos Familiares, realizadas em 2002–2003, 2008–2009 e 2017–2018, indica que alimentos *in natura* ou minimamente processados perderam espaço para alimentos processados e, sobretudo, para os alimentos ultraprocessados^6^. Em Pelotas, dados alimentares dos participantes das coortes de nascimentos de 1982, 1993 e 2004, mostraram que a contribuição energética diária dos alimentos ultraprocessados foi maior na coorte mais jovem (25,1%, 29,8% e 33,7% aos 30, 22 e 11 anos, respectivamente)^7^.

Um estudo realizado com crianças de 6 a 12 meses em uma cidade da região metropolitana de São Paulo, avaliando o consumo de alimentos ultraprocessados nas últimas 24h anteriores à entrevista, mostrou que a prevalência de consumo desses alimentos foi de 43% e que as crianças que não estavam sendo amamentadas, bem como as crianças filhas de mães de menor escolaridade, apresentaram maior prevalência de consumo desses alimentos^8^. Outro estudo, realizado com crianças até 72 meses de idade da cidade de Pelotas-RS, mostrou que a média de consumo energético diário foi de 1.725 kcal/dia e que a participação calórica proveniente do grupo de alimentos ultraprocessados foi de 19,7% nas crianças com menos de 24 meses e 36,1% naquelas com 24 meses ou mais^9^. Porém, dados de consumo de alimentos ultraprocessados por crianças com idade pré-escolar ainda são incipientes.

Os primeiros dois anos de vida são fundamentais para o incentivo e a adoção de hábitos alimentares saudáveis e para prevenção de doenças crônicas em fases posteriores da vida, visto que os hábitos alimentares estabelecidos nessa fase da vida tendem a se manter e se consolidar na vida adulta^10^. Por isso é importante avaliar o consumo de alimentos ultraprocessados por crianças que estão na fase de introdução de alimentação complementar, utilizando uma classificação que não considera somente os nutrientes de um alimento, mas também o seu grau de processamento, contribuindo tanto para uma melhor compreensão dos fatores associados ao consumo de alimentos ultraprocessados quanto com dados que podem auxiliar na elaboração de materiais com orientações sobre práticas alimentares, como o novo Guia Alimentar para Crianças Menores de 2 Anos (2019)^5^, que considera o processamento dos alimentos nas recomendações alimentares para a faixa etária.

Diante disso, o objetivo deste estudo foi avaliar o consumo habitual de alimentos ultraprocessados por crianças de Pelotas-RS aos 24 meses de idade, pertencentes à Coorte de Nascimentos de 2015 e os fatores demográficos, socioeconômicos e comportamentais associados a esse consumo.

## MÉTODOS

### Tipo de Estudo e Amostra

O presente estudo é um corte transversal da Coorte de Nascimentos de 2015, que é um estudo longitudinal de todos os nascidos vivos, realizado em Pelotas, cidade de médio porte do sul do Brasil. As 4.333 crianças, filhas de mães residentes na área urbana da cidade, eram elegíveis para participar do estudo. Descontando uma taxa de perdas e recusas de 1,3% e 54 natimortos, a amostra final da Coorte de Nascimentos de 2015 foi formada por 4.275 crianças. Para a consulta de acompanhamento perinatal, as entrevistas foram realizadas entre 24 e 48 horas após o parto. Posteriormente, as mães e crianças foram entrevistadas em diversas ocasiões (3, 12, 24 e 48 meses de idade da criança). A taxa de acompanhamento da Coorte de Nascimentos de 2015 aos 24 meses de idade foi de 95,4% (n = 4.014). Mais informações encontram-se disponíveis no perfil da Coorte 2015^11^.

### Variáveis

O desfecho do presente estudo, “consumo habitual de alimentos ultraprocessados”, foi avaliado aos 24 meses de idade e coletado por meio de um questionário contendo 20 questões relacionadas à alimentação da criança, a partir da seguinte pergunta: “ *Agora vou fazer algumas perguntas sobre a alimentação do(a) <CRIANÇA>. Por favor responda com base nos alimentos que são consumidos habitualmente, ou seja, todos ou quase todos os dias. Pensando no consumo habitual do(a) <CRIANÇA>, ele(a) toma/come”* . À pergunta, a mãe deveria responder sim ou não.

O questionário de consumo alimentar utilizado no acompanhamento de 24 meses da Coorte de Nascimentos de 2015 foi construído com base no formulário de marcadores de consumo alimentar do Sistema de Vigilância Alimentar e Nutricional^12^. Algumas adaptações foram feitas, a partir do conhecimento empírico sobre as coortes de nascimento e os hábitos de consumo dos pelotenses, principalmente em relação aos exemplos dos itens.

Os itens alimentares contidos no questionário foram classificados de acordo com a classificação NOVA^4^e os alimentos ultraprocessados foram separados em nove grupos: macarrão instantâneo; refrigerantes; suco de caixinha ou engarrafado, suco em pó ou água de coco em caixinha; nuggets, hambúrguer ou embutidos, como presunto, mortadela, salame, linguiça e salsicha; salgadinhos de pacote (tipo chips); iogurte; biscoitos doces ou bolachas recheadas; balas, pirulitos, chicletes, chocolates ou gelatina; e achocolatado adicionado ao leite. O desfecho foi definido como a somatória de alimentos ultraprocessados consumidos habitualmente, variando de zero a nove.

As variáveis independentes avaliadas foram “sexo da criança”, “cor da pele da mãe autorreferida (branca, preta, parda)”, “renda familiar em moeda brasileira” (coletada como somatória das rendas individuais de todos os moradores da casa e logo categorizada em quintis), “idade da mãe em anos” (categorizada menor de 20, 20–34 e ≥ 35), “escolaridade materna em anos completos de estudos” (categorizada como 0– 4, 5–8, 9–11 e ≥ 12), “criança mora com irmãos” (não/sim), foram coletadas no acompanhamento perinatal; já o “status de amamentação da criança” (desmamadas ou parcialmente desmamadas), foi coletado no acompanhamento dos 12 meses, e a variável “criança frequenta creche (não/sim)”, coletada aos 24 meses^a^.

### Análise de Dados

Foi realizada análise descritiva por frequência absoluta (n) e relativa (%) dos alimentos ultraprocessados consumidos e das variáveis independentes para caracterizar a amostra estudada. Também, é apresentada a somatória de consumo habitual de alimentos ultraprocessados aos 24 meses. A associação entre o número de alimentos ultraprocessados consumidos habitualmente e as variáveis independentes foi estimada com análises brutas e ajustadas por regressão de Poisson. Riscos relativos e seus respectivos intervalos de confiança de 95% (IC95%) foram calculados para cada preditor apresentado. As análises foram realizadas a partir de um modelo hierárquico em três níveis, sendo o primeiro nível composto pelas variáveis sexo, cor da pele, idade da mãe, escolaridade da mãe e renda familiar; o segundo nível composto pelas variáveis frequentar creche e ter irmãos; e o terceiro nível composto pela variável status de amamentação. As variáveis independentes, pertencentes a um mesmo nível foram inseridas ao mesmo tempo na regressão. Foram mantidas no modelo ajustado as variáveis que apresentaram valor p < 0,20 e, assim, foram sendo adicionadas as demais variáveis para cada nível. Para a significância estatística foi considerado p < 0,05. As análises foram realizadas no software Stata 15.1.

### Aspectos Éticos

O protocolo do estudo foi revisado e aprovado pelo comitê de ética em pesquisa da Escola Superior de Educação Física, da Universidade Federal de Pelotas (número de registro CAAE: 26746414.5.0000.5313) e a assinatura do Termo de Consentimento Livre e Esclarecido (TCLE) foi obtida antes de cada entrevista.

## RESULTADOS

Um pouco mais da metade da amostra foi composta por meninos (50,6%), em aproximadamente dois terços da amostra as mães eram de cor da pele branca (70,9%) e tinham idade entre 20 e 34 anos (70,6%). Um terço das mães tinham entre 9 e 11 anos completos de estudo (34,1%) e um pouco mais da metade das crianças deste estudo (50,9%) eram filhas únicas. Aos 12 meses de idade, 58,8% das crianças já eram desmamadas e mais de dois terços das crianças não iam para a creche aos 24 meses. A Tabela 1 descreve as características demográficas, socioeconômicas e comportamentais da amostra.

**Tabela 1:** Descrição da amostra segundo características sociodemográficas e obesidade. Coorte de Nascimentos de Pelotas-RS, 2015.

Variável	n (%)
Sexo	
Feminino	2.111 (49,4)
Masculino	2.164 (50,6)
Cor da pele da mãe	
Branca	3.024 (70,9)
Preta	667 (15,6)
Parda	551 (12,9)
Renda familiar^a^	
Q1 (mais pobres)	846 (19,8)
Q2	859 (20,1)
Q3	853 (19,9)
Q4	856 (20,0)
Q5 (mais ricos)	859 (20,1)
Escolaridade da mãe	
0–4 anos de estudo	391 (9,2)
5–8 anos de estudo	1.095 (25,6)
9–11 anos de estudo	1.458 (34,1)
≥ 12 anos de estudo	1.330 (31,1)
Idade da mãe	
≤ 20 anos	623 (14,6)
20–34 anos	3.018 (70,6)
≥ 35 anos	633 (14,8)
Criança frequenta creche aos 24 meses	
Não	2.940 (73,3)
Sim	1.071 (26,7)
Criança tem irmão(s)	
Não	2.174 (50,9)
Sim	2.100 (49,1)
Status de amamentação até os 12 meses	
Amamentação parcial	1.653 (41,2)
Desmamados	2.359 (58,8)

Q: quintil; m: média; DP: desvio padrão.

^a^Renda familiar: Q1 (m = 725,17; DP = 290,42); Q2 (m = 1.369,10; DP = 178,70); Q3 (m = 2.038,80; DP = 211,64); Q4 (m = 3.011,86; DP = 379,93); Q5 (m = 8.133,88; DP = 7.702,16).

A Figura mostra a somatória de alimentos ultraprocessados consumidos aos 24 meses de idade e, na Tabela 2 , é apresentada a prevalência de consumo habitual de cada um dos grupos de alimentos ultraprocessados. Observa-se que 4,6% das crianças consumiram habitualmente os nove grupos desses alimentos. A média de consumo habitual de AUP aos 24 meses de idade foi 4,8 alimentos e a mediana foi cinco alimentos. (DP = 2,3; P25: 3; P75: 7). A prevalência de consumo habitual em cada categoria de alimentos ultraprocessados variou de 29,6% até 88,3%. O alimento menos consumido foi o macarrão instantâneo, seguido por refrigerantes e o alimento ultraprocessado mais consumido foi o iogurte.

**Figura:** Frequência (%) de consumo habitual de alimentos ultraprocessados aos 24 meses de idade. Coorte de Nascimentos de Pelotas-RS, 2015.

**Tabela 2:** Prevalência de consumo habitual de alimentos ultraprocessados aos 24 meses de idade. Coorte de Nascimentos de Pelotas-RS, 2015.

Alimentos ultraprocessados	n (%)
Suco de caixinha ou engarrafado, suco em pó ou água de coco em caixinha	2.641 (65,8)
Refrigerante	1.498 (37,4)
Macarrão instantâneo	1.188 (29,6)
Nuggets, hambúrguer ou embutidos	1.728 (43,1)
Salgadinho de pacote (tipo chips)	1.844 (46,0)
Iogurte	3.542 (88,3)
Biscoitos doces ou bolachas recheadas	2.587 (64,5)
Balas, pirulitos, chicletes, chocolates ou gelatina	2.582 (64,4)
Achocolatado adicionado ao leite	1.724 (43,0)

A Tabela 3 mostra a associação entre consumo habitual de alimentos ultraprocessados e possíveis fatores de risco aos 24 meses de idade. As variáveis “cor da pele”, “renda familiar”, “escolaridade da mãe”, “idade da mãe”, “frequentar creche” e “presença de irmãos” apresentaram associação na análise bruta, o que se manteve na análise ajustada, com exceção da variável “criança frequenta creche”, que perdeu a associação após ajuste para possíveis fatores de confusão ( Tabela 3 ).

**Tabela 3:** Consumo habitual de alimentos ultraprocessados e fatores associados aos 24 meses de idade. Coorte de Nascimentos de Pelotas-RS, 2015.

	Bruta			Ajustada^a^		
	
	RR	p	IC95%	RR	p	IC95%
Sexo		0,686			0,919	
Masculino	0,99		0,97–1,03	1,00		0,97–1,03
Feminino	ref		-	ref		-
Cor da pele da mãe		< 0,001			< 0,001	
Branca	ref		-	ref		-
Preta	1,26		1,21–1,30	1,12		1,08–1,16
Parda	1,21		1,16–1,26	1,07		1,03–1,12
Renda familiar		< 0,001			< 0,001	
Q1 (mais pobres)	1,67		1,59–1,75	1,19		1,12–1,26
Q2	1,54		1,47–1,62	1,17		1,11–1,23
Q3	1,44		1,37–1,51	1,16		1,10–1,22
Q4	1,34		1,27–1,40	1,15		1,09–1,21
Q5 (mais ricos)	ref		-	ref		-
Escolaridade da mãe (anos)		< 0,001			< 0,001	
0–4	1,83		1,74–1,92	1,59		1,50–1,69
5–8	1,69		1,62–1,75	1,47		1,40–1,54
9–11	1,44		1,39–1,50	1,31		1,26–1,37
≥ 12	ref		-	ref		-
Idade da mãe (anos)		< 0,001			< 0,001	
< 20	1,54		1,46–1,62	1,23		1,17–1,30
20–34	1,22		1,16–1,27	1,14		1,09–1,19
≥ 35	ref		-	ref		-
Criança frequenta creche		< 0,001			0,429	
Não	1,16		1,12–1,20	0,99		0,95–1,02
Sim	ref		-	ref		
Criança tem irmão(s)		< 0,001			< 0,001	
Não	ref		-	ref		-
Sim	1,11		1,08–1,14	1,10		1,06–1,13
Status de amamentação até os 12 meses		0,242			0,104	
Amamentação parcial	ref		-	ref		-
Desmamados	1,02		0,99–1,05	1,02		1,00–1,06

RR: risco relativo; IC95%: intervalo de confiança de 95%.

^a^Ajuste por nível hierárquico: nível 1: sexo, cor da pele da mãe, renda familiar, escolaridade da mãe e idade da mãe; nível 2: criança frequenta creche e criança tem irmão (s); nível 3: status de amamentação até os 12 meses.

O número médio de alimentos ultraprocessados consumidos por crianças filhas de mães de cor da pele preta e parda foi, respectivamente, 12% e 7% maior, comparados com o número médio de alimentos ultraprocessados consumidos por crianças filhas de mãe de cor da pele branca. A renda familiar apresentou associação com o consumo habitual de alimentos ultraprocessados, mostrando que o risco de consumo desses alimentos é maior em crianças de famílias do quintil de renda mais pobre (RR = 1,19; IC95% 1,12–1,26). A escolaridade da mãe apresentou uma tendência negativa de associação com o consumo habitual de alimentos ultraprocessados, mostrando que os filhos de mães menos escolarizadas, apresentam maior risco de consumo habitual de alimentos ultraprocessados (RR = 1,59; IC95% 1,50–1,69). Da mesma forma, observou-se que quanto menor a idade da mãe, maior o número médio de alimentos ultraprocessados consumidos. As crianças que possuem irmãos, apresentaram um número médio de alimentos ultraprocessados consumidos 10% maior do que as crianças que são filhas únicas.

## DISCUSSÃO

Este estudo avaliou o consumo habitual de alimentos ultraprocessados aos 24 meses de idade de crianças pertencentes à Coorte de Nascimentos de 2015, de Pelotas-RS e seus fatores demográficos, socioeconômicos e comportamentais associados à este consumo. O elevado número médio de alimentos ultraprocessados consumidos e a introdução precoce de AUP na alimentação das crianças, encontrada em nosso estudo, é corroborada pela literatura, embora os métodos de avaliação variem entre os estudos^3 , 8^. Giesta et al.^3^(2017), verificaram que apenas 21% de uma amostra de crianças de quatro a 24 meses da cidade de Porto Alegre-RS ainda não havia consumido nenhum tipo de AUP, sendo que 56% consumiu algum desses alimentos antes dos seis meses de idade. Relvas et al.^8^(2019), observaram que 43% de uma amostra de crianças de seis a 12 meses de idade de uma cidade da região metropolitana de São Paulo-SP haviam ingerido pelo menos um tipo de alimento ultraprocessado nas últimas 24h. Além da praticidade, conveniência e da alta palatabilidade, a exposição do público infantil aos níveis de marketing que destacam alimentos calóricos, como os ultraprocessados, tem contribuído para trabalhar a lembrança e familiaridade dos alimentos e marcas, por meio de rótulos e embalagens, que podem incentivar e influenciar a compra e consumo dos mesmos^13^.

O consumo excessivo de alimentos ultraprocessados está consistentemente associado a uma deterioração geral da qualidade nutricional das dietas, além de favorecer o consumo excessivo de calorias^14^. Há ainda evidências de que a exposição precoce a edulcorantes (tanto calóricos, quanto não calóricos), utilizados na elaboração de alimentos ultraprocessados, pode causar efeitos adversos na composição corporal, na saúde cardiometabólica e na microbiota intestinal das crianças, além de gerar a preferência por sabor doce, influenciando, assim, o padrão alimentar desde a infância até a idade adulta^15^. Dessa forma, os dados encontrados em nosso estudo são alarmantes, levando em conta as fortes evidências de que alimentos ultraprocessados devem ser evitados nos primeiros anos de vida e consumidos com restrições em todas as outras fases, favorecendo a formação de hábitos alimentares saudáveis e, consequentemente, a prevenção contra doenças crônicas não transmissíveis^10 , 14^.

Nas últimas décadas, houve um aumento do consumo de alimentos ultraprocessados pelos grupos familiares no Brasil. Dados das três últimas edições da Pesquisa de Orçamentos Familiares^6^observaram que os AUP representavam 12,6% das calorias ingeridas diariamente em 2002–2003, chegaram a 16% em 2008–2009 e, na última edição, em 2017–2018, representaram 18,4% do total de calorias. Os hábitos alimentares são influenciados por fatores biológicos, nutricionais, culturais e socioeconômicos e, em crianças, além desses fatores, a alimentação também é influenciada pelos hábitos dos pais e do grupo familiar^16^. Assim, com a crescente participação dos alimentos ultraprocessados na alimentação dos brasileiros, é comum que esse tipo de alimento seja introduzido precocemente na alimentação das crianças, repercutindo nas práticas alimentares e no estado de saúde e nutrição ao longo da vida.

Entre as crianças da Coorte de Nascimentos de 2015, aquelas pertencentes às famílias mais pobres e com menor escolaridade apresentaram maior risco de consumo habitual de alimentos ultraprocessados. Dados nacionais mostram que o aumento do consumo desses alimentos acometeu todos os estratos sociais, entretanto, houve uma tendência de aumento maior entre as classes com menor renda^6^. Em países em desenvolvimento, como o Brasil, a transição nutricional geralmente manifesta-se primeiro nos indivíduos de maior posição socioeconômica. Entretanto, o avanço do desenvolvimento e a progressão da transição nutricional, possibilita maior acesso aos alimentos considerados não saudáveis a grupos de menor posição socioeconômica^17^. Em nosso estudo, as crianças de cor de pele preta e parda apresentaram maior número médio de alimentos ultraprocessados consumidos, comparadas às crianças de cor de pele branca. Considerando que a cor da pele é um marcador de nível econômico, esse fato pode estar ligado ao aumento do acesso da população de menor posição socioeconômica aos AUP, visto que, além de ter maior durabilidade, esses alimentos geralmente apresentam menor custo, comparados aos alimentos frescos, como carnes, frutas e hortaliças^18^. Aliado à isso, os AUP são alimentos de maior praticidade, não necessitando de nenhuma ou quase nenhuma preparação, e podem ser consumidos há qualquer hora, em qualquer lugar, o que contribui para sua preferência na hora da compra^19^. Nesse sentido, ressalta-se a importância das políticas públicas como o Programa Nacional de Alimentação Escolar^20^, cujo objetivo é oferecer alimentação e promover ações de educação alimentar e nutricional a todos os escolares do sistema de educação básica pública, buscando suprir as deficiências nutricionais e alimentares das crianças que não têm acesso à uma alimentação de qualidade.

A literatura aponta, ainda, que, além da menor renda e do baixo nível de escolaridade das mães, a presença de irmãos na composição familiar também é um preditor socioeconômico de uma dieta rica em alimentos ultraprocessados^21 , 22^. Dentre as crianças da Coorte de Nascimentos de 2015, as que possuíam irmãos apresentaram maior número médio de AUP consumidos, comparadas às crianças que são filhas únicas.

O consumo habitual de alimentos ultraprocessados se mostrou associado à menor escolaridade materna, associação relatada em estudos anteriores^8 , 23^. Uma pesquisa nacional identificou associação entre a alta frequência de consumo de alimentos não saudáveis entre crianças menores de um ano e o baixo nível de escolaridade materna^23^. Do mesmo modo, Relvas et al.^8^(2019) apontaram que o maior consumo de alimentos ultraprocessados por crianças entre seis e 12 meses de idade, foi observado entre aquelas cujas mães possuíam menor nível de escolaridade. A idade materna apresentou uma associação negativa com o consumo habitual de alimentos ultraprocessados, visto que as crianças filhas de mães mais jovens apresentaram maior risco de consumo habitual de alimentos ultraprocessados, corroborando achados da literatura^24 , 25^que mostram que as mães mais jovens tendem a introduzir precocemente esse tipo de produto na alimentação das crianças.

O menor acesso às informações sobre práticas alimentares consideradas saudáveis, seja por meio da falta de conhecimento seja pela falta de orientações profissionais no primeiro ano de vida da criança, possivelmente explica a associação da baixa escolaridade materna e da menor idade da mãe com maior risco de consumo de AUP. Evidências^3 , 26^apontam que intervenções nutricionais são efetivas na redução do consumo de AUP, independentemente da idade e escolaridade maternas, quando realizadas com lactentes no primeiro ano de vida, através de orientações nutricionais baseadas nos dez passos da alimentação saudável para crianças brasileiras menores de dois anos^5^. A Estratégia Amamenta e Alimenta Brasil^27^, que reforça e incentiva a promoção do aleitamento materno e da alimentação complementar saudável no Sistema Único de Saúde, é uma importante contribuição para o incentivo e disseminação de informações sobre a importância da alimentação saudável nessa fase da vida. O novo Guia Alimentar para Crianças Brasileiras Menores de 2 Anos^5^, que traz informações sobre o grau de processamento dos alimentos e orientações para promover uma alimentação saudável nos dois primeiros anos de vida, é também um instrumento orientador de políticas, programas e ações que visem apoiar, proteger e promover a saúde e segurança alimentar e nutricional das crianças brasileiras, auxiliando, assim, na diminuição do consumo de alimentos ultraprocessados entre os seis e os 24 meses de idade.

Nosso estudo apresenta algumas limitações. O instrumento utilizado para a coleta de dados alimentares não permite uma estimativa de consumo energético de forma individual, visto que não sabemos a quantidade de alimentos ultraprocessados consumidos, pois o questionário alimentar utilizado na Coorte de Nascimentos de 2015 é qualitativo e não quantitativo. Assim, a partir do instrumento utilizado, não foi possível realizar o ajuste do consumo de AUP por energia. Porém, alguns inquéritos de saúde nacional como a Pesquisa Nacional de Saúde (PNS), Pesquisa Nacional de Demografia e Saúde (PNDS) e Vigilância de Fatores de Risco e Proteção para Doenças Crônicas por Inquérito Telefônico (Vigitel), utilizam uma medida similar à utilizada neste estudo, cujos questionários também avaliam somente a frequência do consumo alimentar e não sua quantidade. O questionário alimentar utilizado na Coorte de Nascimentos de 2015, embora tenha sido construído com base em instrumento conhecido, como o do Sistema de Vigilância Alimentar e Nutricional^12^, não foi validado para as crianças de 24 meses de Pelotas-RS. Por outro lado, um dos pontos fortes deste trabalho é o fato dos dados serem de um estudo de coorte de base populacional, com um grande tamanho amostral e um grande índice de acompanhamento das crianças até os 24 meses de vida, permitindo que os resultados aqui encontrados sejam generalizados para cidades de médio porte. Além disso, o presente estudo contribui para melhor compreensão dos fatores associados ao consumo de alimentos ultraprocessados, colaborando para as políticas públicas voltadas à alimentação e nutrição, como Programa Nacional de Alimentação Escolar e guias alimentares.

Este estudo mostrou que a média de consumo habitual de alimentos ultraprocessados por crianças da Coorte de Nascimentos de 2015 da cidade de Pelotas-RS é elevada e que o risco de consumo desses alimentos foi maior entre crianças filhas de mães de baixa escolaridade, mais jovens e de baixa renda. Segundo as recomendações do Guia Alimentar para Crianças Brasileiras Menores de 2 Anos^5^, o consumo de AUP deve ser evitado nessa faixa etária, visto que esses alimentos são caracterizados como nutricionalmente desbalanceados, além de possuírem um alto teor calórico, grande quantidade de gordura saturada e sódio, baixo teor de fibras e proteínas, e por conterem muitos aditivos, o que pode causar um efeito adverso na saúde das crianças.

Nossos resultados reforçam a necessidade de ações de medidas preventivas e ações de educação alimentar e nutricional voltadas à mães de baixa escolaridade, jovens e de baixa renda e aos profissionais de saúde responsáveis por orientações nutricionais, visto que a infância é um importante período para o incentivo e desenvolvimento de práticas alimentares saudáveis. Ainda são insuficientes os estudos sobre o consumo de alimentos ultraprocessados em crianças com idade pré-escolar. Nesse sentido, sugere-se mais pesquisas sobre os determinantes deste consumo e sobre intervenções para modificar este cenário.

## Footnotes

a Os questionários utilizados em cada acompanhamento da Coorte de Nascimentos de Pelotas 2015 estão disponíveis em: http://epidemio-ufpel.org.br/site/content/coorte_2015/questionarios.php .
